# The Effect of Three Months of Aerobic Training on Stroop Performance in Older Adults

**DOI:** 10.1155/2012/269815

**Published:** 2012-12-11

**Authors:** David Predovan, Sarah A. Fraser, Mélanie Renaud, Louis Bherer

**Affiliations:** ^1^Centre de Recherche, Institut universitaire de gériatrie de Montréal, Montréal, QC, Canada H3W 1W5; ^2^Département de Psychologie, Université du Québec à Montréal, Montréal, QC, Canada H3C 3P8; ^3^PERFORM Centre and Department of Psychology, Concordia University, Montréal, QC, Canada H4B 1R6

## Abstract

Growing evidence supports the use of physical training interventions to improve both physical and cognitive performances in healthy older adults. Few studies have examined the impact of aerobic exercise on Stroop task performance, a measure of executive functions. In the current 3-month aerobic training study, 50 older adults (mean age = 67.96 ± 6.25 years) were randomly assigned to either a three-month physical training group or to a control group (waiting list). Training sessions were 3 times per week for 60 minutes. All participants completed pre- and post-test measures of cognitive performance using the modified Stroop task and physical performance (Rockport one-mile test). Compared to controls, the training group showed significant improvements in physical capacity (*P* < 0.001) and enhanced Stroop performance, but only in the inhibition/switching condition (*P* < 0.03). Furthermore, the increase in aerobic capacity induced by the training regimen correlated negatively with reaction time in the inhibition/switching condition of the Stroop task at posttest (*r* = −0.538; *P* = 0.007). Importantly, the reported gains in cognitive performance were observed after only three months of physical training. Taken together, the results suggest that even short-term physical interventions can enhance older adults' executive functions.

## 1. Introduction

A variety of executive function processes sustained by the prefrontal cortex decline over time [1] (e.g., response preparation [2] and task switching [3]). Recent accounts of executive functions suggest that they rely on distinct basic mechanisms [4], namely, updating (of new information), inhibition (suppressing prepotent responses) and shifting (from one mental set to another), that could be differentially altered as we age. The variability in the trajectories of cognitive decline [5] suggests that compensatory mechanisms [6] and individual factors (i.e., involvement in physical activity and cognitive reserve) [7] could minimize these deficits.

Physical activity, defined as any activity that involves bodily movements, is one individual factor that can reduce the impact of aging on executive functions [8, 9]. In goal-directed research, physical activity that was planned, structured, and repetitive improves physical fitness, defined as the ability to function efficiently and effectively in work and leisure activities and to meet unforeseen emergency situations [10]. Theoretically, the “selective improvement” hypothesis [11] argues that aerobic exercise known to increase cardiorespiratory fitness as indexed by direct measures or estimations of *V*_O_2_max⁡_ (i.e., capacity of the body to transport and use oxygen during incremental exercise) should present the largest benefit in tasks requiring executive control processes [12, 13] (but also see [14] for the effect of resistance training).

A recent review [15] found evidence that long-term physical activity interventions have consistent effects on tasks requiring inhibition or requiring dual-task coordination, compared to tasks requiring shifting which suggests that basic executive function mechanisms may be differentially affected by physical activity. However, the consistency of the effect on dual-task coordination remains precarious as it is based on only two results [16, 17]. In the current study, we explore the effect of aerobic exercise on a complex task requiring both inhibition and shifting (dual executive function condition), compared to a task requiring only inhibition.

One task commonly used to explore age differences in inhibitory control processes is the Interference condition of the Stroop color-word task [18]. In this condition, the Stroop effect occurs when naming the color of the ink in which a word is written, and this happens faster and more accurately when the color denoted by the name is congruent (e.g., blue written in blue), rather than incongruent (e.g., blue written in red). When compared to younger adults, older adults demonstrate longer reaction times (RTs) and higher error rates in the incongruent condition (i.e., interference condition) [19–22].

The present study targeted sedentary older adults (i.e., no or irregular physical activity) and examined if the benefits of aerobic exercise extend to different executive processes measured by using a modified version of the Stroop task [23]. This modified version includes a switching condition in addition to the classic interference or inhibition condition. The executive component of shifting, or the ability to switch between different task sets, has demonstrated the most robust age-related deficits [24] and has been identified as an important predictor of the maintenance of independent living in elderly [25]. In accordance with Barenberg et al. [15], the aerobic exercise training benefit on executive functions should be observed in the inhibition condition. In comparison to the inhibition condition, we expect the dual executive function condition to demonstrate smaller training benefits, since the existing findings of long-term physical activity benefits on tasks requiring shifting are less consistent [15].

## 2. Methods

### 2.1. Participants

Through advertisement in community centers in the Montreal area, 77 older adults were recruited. All participants completed a phone screening evaluating their physical health prior to admission in the study. The level of risk when engaging in intense physical activity and the level of physical activity over the 12 past months were assessed by the completion of the modified questionnaire of aptitude to physical activity (QAA-P) and the modifiable activity questionnaire (MAQ) [26]. Only sedentary adults, whose frequency of physical activity was less than twice per week, were enrolled.

Exclusion criteria included a history of neurological disease or major surgery in the year preceding the study, auditory or visual impairments that had not been corrected, cardiovascular disease or vascular peripheral attacks, and/or moderate to severe hypertension. In order to exclude participants with dementia or depression, a score lower than 26/30 on the Mini-Mental State Examination (MMSE) [27] and higher than 11 on the Geriatric Depression Scale (GDS) [28] resulted in exclusion. Ten participants did not meet inclusion criteria.

Assignment of the remaining 67 participants was based on the order of recruitment and on participants' willingness to engage in a 3-month fitness training program. Therefore, 32 participants were assigned to the experimental group, and the remaining 35 were assigned to the control group who did not receive any training. After completing the screening, seven participants in the training group and ten in the control group decided not to pursue the study for personal reasons. In the training group, there were no differences in baseline characteristics between the individuals who decided not to pursue and those who remained in the study. In the control group, those who discontinued were older (*P* < 0.05) and had lower scores (*P* < 0.05) on the similarities subtest of the WAIS-III.

The remaining participants in both the aerobic exercise group (F/M = 21/4; age (years) = 67.80 ± 6.60; education (years) = 14.36 ± 4.17) and the control group (F/M = 21/4; age (years) = 67.72 ± 6.01; education (years) = 12.92 ± 2.61) completed the study. Within these 50 participants, one participant in each group was excluded from statistical analyses due to missing Stroop test data. On a five-point health-rating scale (5 = excellent), the experimental (*M* = 4.21, SD = 0.74) and control groups (*M* = 4.17, SD = 0.72) average ratings were equivalent.  Table 1 presents baseline characteristics of both groups.

### 2.2. Procedure

During the pre-test session, the consent form was signed, and all screening tests and questionnaires were completed, along with the modified Stroop task (see description below). In the second session, a test of cardiorespiratory fitness (Rockport one-mile test, [29]) was completed. Then the training group participated in a 12-week aerobic fitness program (see below). In a post-test session, both groups were reevaluated with the same neuropsychological battery (including the Stroop task) and the Rockport one-mile fitness test. The study was completed within a 14-week period (1 week of pre-testing, 12 weeks of training, and 1 week of post-testing).

### 2.3. Aerobic Fitness Program

The 3-month (3 × 1 hour/week) physical exercise program was composed of stretching and cardiorespiratory exercises (fast walking and aerobic dancing). Adequate warm-up and cool-down periods and progressive and gradual increments in exercise duration and energy expenditure were implemented according to recommendations [30]. Duration of cardiorespiratory exercise was also gradually increased during the training program, beginning at 15 min per session and increasing until participants were exercising for 40 min per session. The training sessions were continuously supervised by two professional kinesiologists. Training session attendance was high, with participants attending 86.58% of sessions.

### 2.4. Pre- and Post-Test Measures: Cardiorespiratory Fitness Assessment

The Rockport one-mile test [29] was employed to evaluate cardiorespiratory fitness. This walking test provides an accurate estimation of the maximum level of oxygen consumption (*V*_O_2_max⁡_; [29]). A strong correlation coefficient (*r* = 0.88) has been reported between the Rockport estimated *V*_O_2_max⁡_ and a direct measure of *V*_O_2_max⁡_ during an incremented test on a treadmill [29]. Participants wearing Polar S120 heart rate monitor (Polar Electro, Lake Success, NY, USA) were instructed to walk one mile without stopping, as fast as possible. The time required to complete the distance was manually recorded on a stopwatch. Heart rate frequency was recorded 1 min after the end of the walking test. *V*_O_2_max⁡_ was estimated using the equation provided by [29] that takes into account participants' weight, age, sex, cardiac frequency post-exercise, and time to walk the one-mile distance.

### 2.5. Pre- and Post-Test Measures: Stroop Tasks

The present study utilized a Stroop test [23] that has four different conditions. Each condition included 100 stimuli (10 items per line) printed on a 21.5 × 28 cm sheet of paper. In the first (reading) condition, the participant had to read the words printed in black (i.e., red, green, blue, and yellow). In the second condition (naming), the participant needed to name the color of the rectangles. In the third condition (interference), the participant had to name the color of the ink in which the words are written. The meaning of each word had to be ignored, since it was incongruent with the color to name (e.g., the word “green” written in red). This condition is the inhibition only condition. The last condition (Inhibition/Switching) was similar to the third condition, except that 20 color words out of the 100 items were surrounded by a small box to indicate to the participant to read the word instead of naming its color. Therefore, the participant must alternate (or switch) in the inhibition/switching condition between naming the ink color of the words and reading the words. This is considered the most complex condition as it requires two executive processes (inhibition and switching). For all conditions, participants were to respond as quickly as possible, while making the least amount of errors. Reaction times (RTs) and the number of errors (corrected and uncorrected) were the main variables of interest.

## 3. Results

### 3.1. Participant's Baseline Characteristics

An ANOVA revealed no significant difference between groups on their level of formal education, *F*(1,46) = 2,03, *P* < 0.16, MMSE, *F*(1,46) = 3,26, *P* < 0.08, general verbal ability (score on the similarities subtest of the WAIS-III), *F*(1,46) < 1, GDS, *F*(1,46) < 1, and MAQ score, *F*(1,46) = 1,01, *P* < 0.32. Training and control groups were also comparable for age, *F*(1,46) < 1.

### 3.2. Cardiorespiratory Fitness Assessment

**Figure 1:**
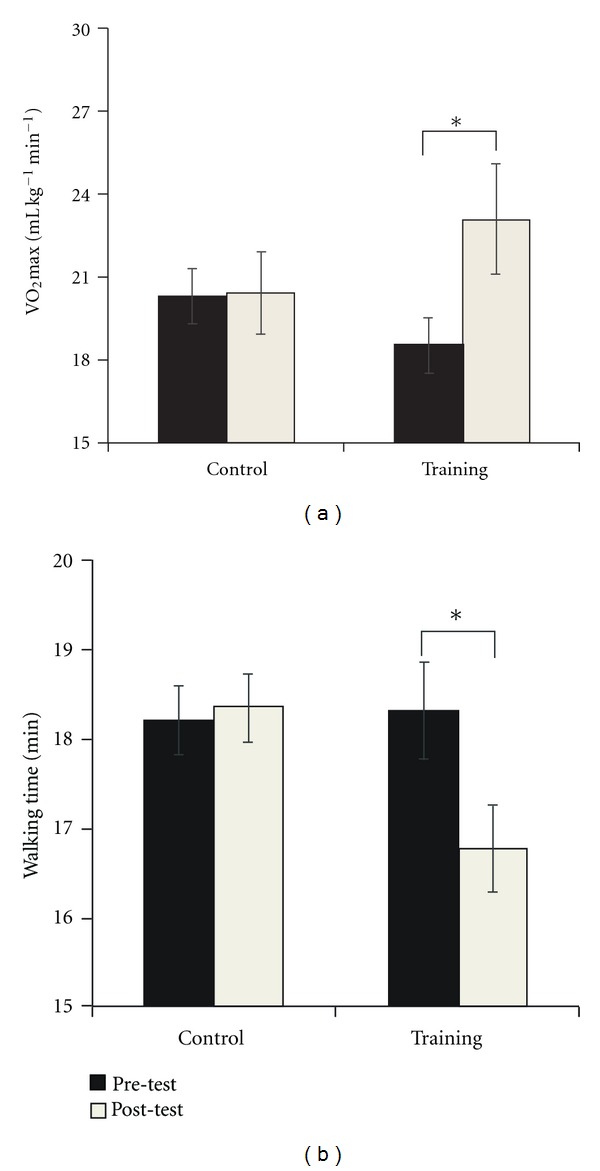
Mean and SE for *V*_O_2_max⁡_ estimate (a) and walking time (b) in the Rockport one-mile test. _ _*P < .001.

A significant group × time interaction was found, *F*(1,44) = 24.99, *P* < 0.001, which confirmed that the participants in the training group showed significant improvement in the *V*_O_2_max⁡_ estimate (see Figure 1(a)) after 3 months of physical fitness training, *F*(1,23) = 39.00, *P* < 0.001, while the *V*_O_2_max⁡_ estimate of control participants remained unchanged, *F*(1,23) < 1. The analysis of walking time (see Figure 1(b)) also confirmed the benefits of the aerobic fitness intervention, as evidenced by a significant group × time interaction, *F*(1,44) = 33.69, *P* < 0.001. Participants in the training group walked the mile faster after the 12-week training program, *F*(1,23) = 44.44, *P* < 0.001, whereas the walk time of participants in the control group did not change significantly, *F*(1,23) < 1.

### 3.3. Stroop Task

**Figure 2:**
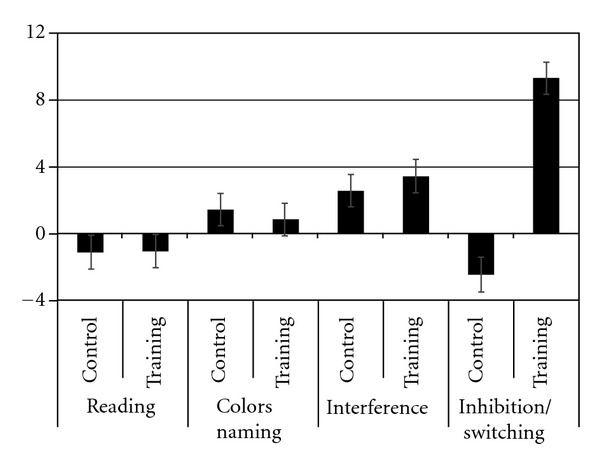
Mean (s) and SE for the change score on the Stroop test. _ _*P < .05.

At baseline, group performance on Stroop tasks (see Table 2) was equivalent as no significant differences were observed on the reading, *F*(1,  46) < 1, naming, *F*(1,46) = 2,54, *P* < 0.19, interference, *F*(1, 46) = 2.37, *P* < 0.13, and inhibition/switching condition, *F*(1,23) < 1. For each Stroop condition, a score measuring the amount of change due to the intervention was computed as follows: pre-test minus post-test score (see Figure 2). An MANOVA revealed a significant effect of group for the inhibition/switching condition, *F*(1,46) = 5.03, *P* < 0.03. No difference was found for the reading, *F*(1,46) < 1, naming, *F*(1,46) < 1, and interference condition, *F*(1,46) < 1. Follow-up univariate analyses on the change score in the inhibition/switching condition confirm the effect of the intervention on the training group, *F*(1,23) = 5.05, *P* < 0.03, and no difference was found for the control group, *F*(1,23) < 1. Furthermore, the increase in aerobic capacity correlates negatively with reaction time at post-test only in the inhibition/switching condition of the Stroop task (*r* = −0.54; *P* = .007) for the training group.

Table 2 presents overall error rates for each condition of the Stroop test. Analyses on error rates using a Wilcoxon signed ranks test showed that for the control and the training group, the amount of corrected errors for the interference and inhibition/switching condition remains equivalent at month 3. The amount of uncorrected errors improved in the control group only for the interference condition (*Z* = −2.10, *P* = 0.04), and the training group improved only for the inhibition/switching condition (*Z* = −2.86, *P* = 0.004).

## 4. Discussion

The results of the present study suggest that, in the elderly, the benefits of an aerobic training program occur primarily on executive functions and that these benefits can be seen after only three months of physical training. As a first step, we confirm based on the Rockport walk test that our trained group demonstrates improved cardiorespiratory fitness when compared to the control group after 12 weeks of physical training. In addition, the training group showed a performance improvement on the Stroop task requiring multiple executive processes, the inhibition/switching condition. As inhibition alone was not improved, we explain this in terms of improvement in the switching domain. One explanation could be the type of exercise done during the training. As previously mentioned, aerobic dance exercise was part of the training our participants received. Research [31] has already supported improved cardiorespiratory endurance after 12 weeks of low-impact aerobic dance in a group of elderly women. In addition, similar to our findings, Coubard et al. [32] evaluated executive functions after contemporary dance training and demonstrated an executive function improvement after 5.7 months of contemporary dance that was seen only in a switching condition (rule shift cards sorting test) but not in an inhibition condition (Stroop task). Together with our results, this suggests that some types of exercise might selectively improve complex tasks involving switching and/or multiple executive processes.

The absence of a specific training effect on the Interference condition is at odds with some studies, including the review of Barenberg et al. [15]. For example, in a 4-month (3 × 1 hours/week) trial, Dustman et al. [33] demonstrated that the Stroop interference condition performance only improved for those aerobically trained. Interestingly, Blumenthal et al. [34] employing an aerobic training of the same duration and intensity did not replicate this finding. The choice of control group activities does not explain these divergent findings as both studies included a group who did not exercise. A possible confounding factor is that both studies computed the Stroop effect differently. In Dustman's study, RTs in the reading condition were subtracted from the “interference” condition, as a way to account for improved motor ability. It has been proposed [22] that these proportional interference scores minimize the effects of general slowing, which is often highlighted as a potential explanation of the magnitude of the Stroop effect in the elderly [35–37]. Therefore, the absence of an aerobic benefit in inhibition in Blumenthal's study and our study could have been hindered by other factors that are not task specific. 

Blumenthal et al. [34] acknowledged two factors that could account for the difference in the findings between their study and that of Dustman et al. [33]: (1) different sample characteristics (age and male/female ratio) and (2) differences in aerobic fitness improvement levels. In fact, Blumenthal et al. [34] reported that their participants experienced less than 50% (11.6% versus 27%) of the improvement in *V*_O_2_max⁡_ reported in Dustman study. In the current study, there were no differences in our two groups based on age or male/female ratio, and we demonstrate a significant improvement in *V*_O_2_max⁡_ in our training group, as such our findings are not confounded by these factors. 

Finally, it is important to note that our results show that the improvement in cardiovascular fitness and in executive performance in our training group is correlated. The increase in cardiorespiratory fitness correlates negatively with reaction time in the inhibition/switching condition of the Stroop at post-test, suggesting that there is indeed a relationship between improved cardiorespiratory fitness and improvements in a Stroop condition requiring two executive processes. We did not find a significant correlation between cardiorespiratory fitness and the inhibition-only performance which is in line with a recent finding that suggests that an improvement in cardiorespiratory fitness is not prerequisite to an enhanced Stroop performance [38]. Smiley-Oyen et al. [38] had equivalent *V*_O_2__ peak improvements in both their aerobic (18%) and strength/flexibility exercise training groups (13%) after completing 10 months (3 × 1 hours/week) of training, and yet Stroop performance as measured by RTs and accuracy improved only in the aerobic group. This outcome highlights the possibility that the impact of aerobic exercise on the Stroop performance might not be directly mediated by changes in cardiorespiratory fitness per se. In light of this possibility, other mechanisms have been proposed such as enhancement of brain vascularization, an increased plasticity though neurogenesis, neurotrophic factors (e.g., brain-derived neurotrophic factor (BDNF)) [39], or other factors that improve neuronal viability [40, 41]. 

Two limitations affect the generalizability of our results. First, the method of assignment was done by self-selection, potentially placing more motivated individuals in our training group; a future study should involve a more systematic and randomized method of assignment. Second, our waiting-list control group was only assessed at pre and post time points, and as such, we cannot control for any effect of socialization that may have occurred in the training group since they came repeatedly over the 12-week period. A better approach would have been to propose a control intervention with the same training regime that includes physical activity but that does not increase cardiorespiratory fitness. 

 Future research is needed to clarify why in the present study participants in the training group did not improve on the Inhibition condition and why other published studies have reported improvement (Liu-Ambrose et al. [14], for instance). One promising avenue would be the use of imaging techniques as they have shown an age-related brain activation difference when performing a Stroop task [42]. Of particular interest, a recent study [43] has found distinct patterns of prefrontal activation between the inhibition condition and the inhibition/switching condition using functional near infrared spectroscopy (fNIRS). Therefore, brain imaging studies could contribute to the comprehension of the condition-related effect of aerobic training in aging that is observed with the Stroop paradigm, particularly in the inhibition/switching condition, known to be sensitive to cognitive decline in the elderly [44–47].

## Figures and Tables

**Table 1:** Participant's baseline characteristics.

	Control	Training
	*M* (SD)	*M* (SD)
General characteristics		
Age	68.08 (5.85)	67.83 (6.74)
Education	12.96 (2.66)	14.42 (4.25)
General mental ability		
MMSE	29.20 (1.14)	28.62 (1.10)
Verbal intelligence		
Similarities	22.87 (4.41)	22.58 (4.89)
Mood assessment		
GDS	5.37 (4.36)	4.50 (2.83)
Fitness assessment		
MAQ	3.44 (2.62)	4.50 (4.45)

**Table 2:** Participant's baseline and follow-up results (Mean (SD)) for the Stroop test.

	Control		Training	
	Before	After	Before	After
Reaction time (s)				
Reading	44.26 (6.87)	45.40 (6.84)	43.82 (6.29)	44.90 (6.45)
Colors naming	71.39 (16.69)	69.97 (15.83)	64.81 (11.40)	63.97 (9.06)
Interference	126.03 (30.39)	123.48 (35.81)	113.57 (25.49)	110.16 (27.41)
Inhibition/switching	132.55 (21.21)	135.04 (29.48)	135.56 (38.94)	126.24 (27.29)
Error				
Reading	0.17 (0.38)	0.08 (0.28)	0.08 (0.28)	0.13 (0.34)
Colors naming	0.92 (1.21)	0.92 (0.88)	1.38 (1.28)	0.75 (0.68)
Interference	2.17 (2.73)	1.71 (1.49)	2.08 (2.21)	1.75 (1.39)
Inhibition/switching	2.50 (2.36)	2.17 (1.83)	4.79 (4.03)	3.00 (3.11)
